# Characterization of Peptide Profiles and the Hypoallergenic and High Antioxidant Activity of Whey Protein Hydrolysate Prepared Using Different Hydrolysis Modes

**DOI:** 10.3390/foods13182978

**Published:** 2024-09-20

**Authors:** Qiang Cui, Yuting Li, Tingli Li, Jie Yu, Guanghui Shen, Xiaomeng Sun, Man Zhou, Zhiqing Zhang

**Affiliations:** 1 College of Food Science, Sichuan Agricultural University, Ya’an 625014, China; cuiqiangwx@163.com (Q.C.); 18882839491@163.com (Y.L.); 13388383453@163.com (T.L.); jieyu0609@163.com (J.Y.); shenghuishen@163.com (G.S.); zhouman@sicau.edu.cn (M.Z.); 2 Key Laboratory of Dairy Science, Northeast Agricultural University, Harbin 150030, China; sunxm@neau.edu.cn

**Keywords:** whey protein isolate, hydrolysis, peptidomics, peptide profiles, bioactive properties

## Abstract

Food proteins and peptides are generally considered a source of dietary antioxidants. The aim of this study was to investigate the antioxidant activity, allergenicity, and peptide profiles of whey protein hydrolysates (WPHs) using different hydrolysis methods. The results demonstrated that the degrees of hydrolysis of the hydrolysates with one-step (O-AD) and two-step (T-AD) methods reached 16.25% and 17.64%, respectively. The size exclusion chromatography results showed that the O-AD had a higher content of >5 and <0.3 kDa, and the distribution of peptide profiles for the two hydrolysates was significantly different. Furthermore, 5 bioactive peptides and 15 allergenic peptides were identified using peptidomics. The peptide profiles and the composition of the master proteins of the O-AD and T-AD were different. The DPPH and ABTS radical scavenging abilities of WPHs were measured, and hydrolysates were found to exhibit a strong radical scavenging ability after being treated using different hydrolysis methods. An enzyme-linked immunosorbent assay showed that the sensitization of WPHs was significantly reduced. This study may provide useful information regarding the antioxidant properties and allergenicity of WPHs.

## 1. Introduction

Whey protein is a high-quality protein source that is easy to digest and absorb, with high bioavailability and nutritional value [1]. It is mainly composed of β-Lactoglobulin (β-Lg), α-Lactalbumin (α-La), bovine serum albumin (BSA), immunoglobulins (IGs), and lactoferrin (LF) [2]. Among them, β-Lg and α-La are the two main globular proteins in whey protein, accounting for 70~80% of the total [3]. Whey protein is a rich source of bioactive peptides, which can play a role in the diet management of chronic diseases [4,5]. It has potential nutritional and health care properties, such as lowering blood pressure, anti-oxidation, and preventing cardiovascular disease [6].

Although whey protein has obvious advantages, it is a common allergen (especially β-Lg and α-La) that usually causes allergic diseases in people. Researchers are committed to modifying whey protein to destroy its structure so as to reduce its allergenicity as much as possible. In general, the modification methods can be divided into two categories: (1) The first is thermal modification. Lamberti et al. [7] evaluated the effects of two heating methods (boiling on a hotplate or in a microwave oven) on milk protein sensitization. The results showed that heating induction produces β-Lg aggregates in high molecular weight products, and there is no immune reaction in the serum of subjects with a milk protein allergy. Domestic boiling modifies the milk protein profile, causing a minor reduction in milk allergenicity. Zhang et al. [8] studied the influence of thermal processing on the potential allergenicity of peanut protein and found that the potential allergenicity of protein changed significantly after thermal processing. The allergenicity increased after baking, while the protein allergenicity decreased after cooking. (2) The second type is non-thermal processing. Yang et al. [9] evaluated the effect of β-Lg on its structure and allergenicity under irradiation and sonication treatments. The results showed that ultrasonic treatment had no significant effect on the sensitization but could change the structure of bovine β-Lg. Cui et al. [10] analyzed the allergenicity and peptide distribution of milk protein hydrolysates. The results of cellular experiments confirmed that milk protein hydrolysates were significantly less allergenic, but there were differences in the level of allergenicity after different enzyme treatments.

Considering the degree of hydrolysis and the protein’s molecular weight, they can be divided into partial hydrolysis (PH) and extensive hydrolysis (EH). The molecular weight of a peptide segment in a partially hydrolyzed formula is generally 3–10 kDa [11]. According to relevant reports, PH contains a large number of peptides greater than 6 kDa [12], which can cause allergic reactions. The highly hydrolyzed formula can significantly reduce the allergenicity of the peptides produced. Although there is no clear definition of EH, EH can be classified according to the molecular weight of peptides, and the molecular weight of almost all peptides in the products of extensive hydrolysis is less than 3 kDa. Considering the possible interaction between proteases, hydrolysis methods are divided into one-step and two-step methods [10,13,14]. There are many factors that affect the outcome of protein hydrolysis, including the time of hydrolysis, the ratio of the enzyme to substrate, and the type of enzyme. However, a major challenge in the production of extensive protein hydrolysates is the identification of the peptide profiles of hydrolysates and residues of allergenic peptides. Peptide information regarding protein hydrolysis products can be characterized using the peptidomics approach. Wang et al. [15] identified the difference in protein profiles when kefir grains from different regions were subcultured in goat milk by using peptidomics technology. The results showed that a larger number of peptides were found from different kefir grains by using peptidomics. Currently, peptidomics is mainly used in protein hydrolysates to determine which peptides are released in the production process.

The team in the previously mentioned research found that a combination of exoproteases and endoproteases can be used to obtain deep milk protein hydrolysates. After determining the released peptide information, we can also identify the potential functional activities. At the same time, it can also be used to monitor the degradation of allergenic food proteins to ensure the elimination of allergenic epitopes [16]. Therefore, the objectives of this study were as follows: (1) analyze the effects of different modes of hydrolysis on the peptide composition; (2) analyze allergens and bioactive peptides in hydrolysis products from different modes to provide guidance for optimizing hydrolysis methods.

## 2. Materials and Methods

### 2.1. Materials

Whey protein isolate (WPI) with 94% purity was purchased from Fonterra Co., Ltd. (Auckland, New Zealand). Alcalase 2.4 L (1 × 10^5^ U/g) was obtained from Novozyme Biotechnology Co., Ltd. (Beijing, China). Protease A 2SD was supplied by Amano Enzyme Inc. (Nagoya, Japan). Additionally, all chemicals used in this study were of reagent grade. Ultrapure water was used in all experiments.

### 2.2. Preparation of WPHs

WPI was dissolved in ultrapure water to prepare a solution of 5 g/100 mL. The hydrolysis conditions were set as follows: Alcalase–Protease A 2SD was named AD. The hydrolysis was carried out at 50 °C with a pH of 8.0. Based on previous studies [10,13,14], in this study we used one-step (enzymatic hydrolysis with the simultaneous addition of both enzymes to a protein solution, O-AD; Alcalase (E/S weight ratio of 1 g:50 g), Protease A 2SD (E/S weight ratio of 1 g:50 g)) and two-step (enzymatic hydrolysis of the two enzymes by adding each to a protein solution, T-AD; Alcalase (E/S weight ratio of 1 g:50 g), Protease A 2SD (E/S weight ratio of 1 g:50 g)) hydrolysis methods. In the one-step hydrolysis method, the hydrolysis time was 2 h. In the two-step hydrolysis method, Alcalase was first hydrolyzed and inactivated in a boiling water bath for 10 min after 2 h. In the second stage, Protease A 2SD was added for hydrolysis, and the hydrolyzed samples were inactivated by heating them in boiling water for 10 min after the same 2 h. Finally, the hydrolyzed samples were separately centrifuged (15 min, 8000× *g*) and then freeze-dried [14]. The next study was carried out according to Figure S1.

### 2.3. Degree of Hydrolysis (DH)

The DH of WPH was measured following the method outlined by Zhang Chen and He [17]. Mix 400 μL of WPH with 3 mL of o-phthalaldehyde (OPA) reagent. Incubate for 5 min at room temperature and measure the absorbance at 340 nm. The DH was calculated using the following equations:

(1) $$\mathrm{Serine}-{\mathrm{NH}}_{2}=\frac{{\mathrm{OD}}_{\mathrm{sample}}-{\mathrm{OD}}_{\mathrm{blank}}}{{\mathrm{OD}}_{\mathrm{standard}}-{\mathrm{OD}}_{\mathrm{blank}}}\times \frac{\left(0.9516\times V\times 100\right)}{\left(X\times P\right)}$$

where V is the sample volume (L), X is the sample weight (g), and P is the protein content (%) of the sample.

(2) $$h=\frac{\left(\mathrm{Serine}-{\mathrm{NH}}_{2}\right)-\beta }{\alpha }$$

where α is 0.97 and β is 0.342.

(3) $$\mathrm{DH}=\frac{h}{h_{\mathrm{tot}}}\times 100$$

where h is the number of hydrolyzed bonds and h_tot_ is 8.8.

### 2.4. SDS-PAGE

A modified method from Cui et al. [18] was used to determine the SDS-PAGE of WPHs. Concentration gels (4.5%) and separation gels (15%) for SDS-PAGE were prepared. The samples (5 mg/mL) were mixed with SDS uploading buffer and boiled; the sample volume was 7 μL, and the voltage was set to 90 V at the beginning of electrophoresis and 120 V when the samples were put into the separation gels. BeyoBlue^TM^; Caulmers Brilliant Blue Ultrafast Staining Solution (Shanghai Beyotime Biotechnology Co., Ltd., Shanghai, China) was used to stain proteins for 1 h. Then, decolorization was performed using ultrapure water.

### 2.5. Molecular Weight Distribution

The molecular weight distribution of WPHs was determined using size exclusion chromatography (TSKgel 2000 SWXL, Tosoh Co., Tokyo, Japan). The mobile phase conditions were set as follows: water/acetonitrile/trifluoroacetic acid (55/45/0.1, *v*/*v*/*v*) [19].

### 2.6. Peptidomics

#### 2.6.1. EASY-nLC1200 Q Exactive Plus

After desalination using C18 stage tips, the WPH samples were measured using EASY-nLC1200 Q Exactive Plus in accordance with the method described by Wang et al. [15]. The samples were sequentially injected into a C18 reverse phase pre-column (100 μm × 2 cm, 5 μm) and a C18 analytical column (75 μm × 100 nm, 3 μm) and eluted through a mobile phase linear gradient (containing phase A, 0.1% formic acid, and phase B, 80% acetonitrile) at a flow rate of 300 nL/min. All samples were subsequently identified via the Q-Exactive system (Thermo Fisher Scientific, Waltham, MA, USA) at 325 °C capillary temperature, 350–2000 m/z scanning range, and 45 ms maximum ion injection time. The analysis time was 120 min. 

#### 2.6.2. Peptide Identification

The mass data of samples were subjected to analysis via Proteome Discoverer software 2.2. The abundance of proteins and peptides in the hydrolysate was determined using a label-free quantification method. The Biopep database was used to find allergens and bioactive peptides (https://www.uwm.edu.pl/biochemia/index.php/pl/biopep, accessed on 24 June 2021, 4321 entries).

### 2.7. Determination of Bioactive Properties

#### 2.7.1. DPPH Radical Scavenging

A 2 mL aliquot of sample solution (0.1–3.0 mg/mL) was mixed with an equal volume of DPPH reagent (0.2 mM, in 95% ethanol) and then reacted for 30 min in the dark. The absorbance of the reactant was recorded at 517 nm, with deionized water as a blank [13].

#### 2.7.2. ABTS Radical Scavenging Activity

The ABTS radical scavenging activity was analyzed according to the method of Silveira Coelho et al. [20]. The hydrolysate (40 µL) solution (0.1–5.0 mg/mL) was mixed with ABTS solution (5 mL, containing 3.5 mM ABTS and 1.225 mM potassium persulfate) and then incubated at room temperature for 6 min in the absence of light. The absorbance of the reactant was recorded at 734 nm.

#### 2.7.3. Determination of In Vitro Antigenicity

The potential allergenicity was examined using pooled serum from Plasmalab International Co., Ltd. (Everett, WA, USA) (Table S1). IgG and IgE binding through indirect competitive ELISA was used to analyze the allergenicity of the WPH sample [8,21].

### 2.8. Statistical Analysis

The experiments were performed with three replications, and measurements were taken in triplicate. One-way analysis of variance (ANOVA) (*p* < 0.05) and Duncan’s test were utilized in SPSS 20 software (SPSS Inc., Chicago, IL, USA) and expressed as mean ± standard deviation. All graphs were drawn by Origin 2021 (Origin Lab Corporation, Northampton, MA, USA).

## 3. Results and Discussion

### 3.1. Degree of Hydrolysis and SDS-PAGE

**Figure 1:**
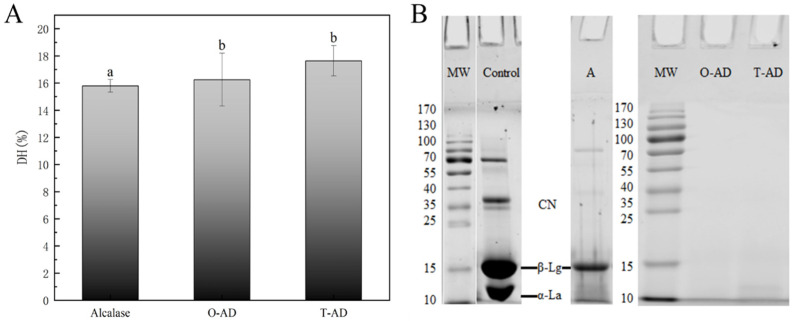
DH (**A**) and SDS-PAGE (**B**) of WPI and WPHs. (MW: standard protein; Control: whey protein isolate; A: Alcalase group; O-AD: Alcalase–Protease A 2SD by one-step; T-AD: Alcalase–Protease A 2SD by two-step). Different letters indicate significant differences between samples of different concentrations (*p* < 0.05).

The essential indicator of the protein hydrolysis reaction is the degree of hydrolysis (DH). This is because the DH marks changes in protein peptide bonds, resulting in the breakdown of intact proteins into smaller peptides or amino acids [22]. The DH of the WPHs produced using two different hydrolysis methods (one-step and two-step) after 2 h is illustrated in Figure 1A; WPI hydrolyzed by alcalase was used as a control group. The results indicated that the DH of WPI reached 15.8% after alcalase hydrolysis. However, the DH of the final hydrolysate was significantly higher than that of the control using a combination of Alcalase and Protease A 2SD to hydrolyze WPI using both one-step and two-step methods (*p* < 0.05). Alcalase is an endopeptidase with high efficiency for hydrolysis. This occurs mainly because of its extensive hydrolysis sites, mainly the hydrolysis of aromatic amino acid residues. Furthermore, the enzyme exhibited a high degree of specificity for aromatic, acidic, sulfur-containing, aliphatic (Leu and Ala), hydroxyl-containing, and basic residues [23]. Similarly, Protease A 2SD had high exopeptidase and endopeptidase activities with a wide range of hydrolysis sites. This confirmed that combining exopeptidases and endopeptidases could significantly improve the hydrolysis efficiency. These results were consistent with the findings of Kamnerdpetch et al.’s [24] study on potato pulp protein hydrolysate, in which the researchers discovered that hydrolysis with a combination of alcalase (2%) and flavourzyme (5%) resulted in hydrolysates with DH values as high as 44%.

Most enzymes are also proteins. Considering that interactions might exist between proteases, the hydrolysis methods were categorized into one-step and two-step methods. Although a combination of endopeptidase and exopeptidase could significantly improve the hydrolysis efficiency, the findings showed that the DH of the hydrolysate differed among different hydrolysis methods. This outcome was not surprising because the pattern of protein degradation was different for the same substrate when treated using different hydrolysis methods. This disparity mainly stemmed from variations in the enzymatic cleavage sites, eventually leading to differences in their bioactivity or functional properties. Therefore, it is necessary to understand the peptide profiles of hydrolysates in different hydrolysis modes.

As shown in Figure 1B (SDS-PAGE), the main fractions of BSA, casein, α-La, and β-Lg are present in WPI. This is mainly due to the fact that other components (BSA, casein, etc.) in the milk cannot be removed entirely during membrane separation or other processes to produce WPI. After hydrolysis by alcalase, the BSA, casein, and α-La fractions disappeared, and their peptide molecular weights (MWs) were all <10 kDa. However, Figure 1B shows the incomplete hydrolysis of β-Lg. The SDS-PAGE results were similar to the observation of Banach, Lin, and Lamsal [25], who found that β-Lg was more resistant to hydrolysis by endopeptidases than casein due to its compact globular structure. WPI was completely hydrolyzed following treatment using two different hydrolysis methods (one-step and two-step methods), and the results showed that the MWs were <10 kDa for all peptides in the WPHs. The bioactive peptides in WPI might be released, and their allergenicity might also change with a decrease in MW. Therefore, we continued to measure WPHs using the volume exclusion technique to determine the MW range and reveal the effect of different hydrolysis methods on the bioactivity and sensitization of WPI hydrolysates.

### 3.2. Molecular Weight Distribution

**Figure 2:**
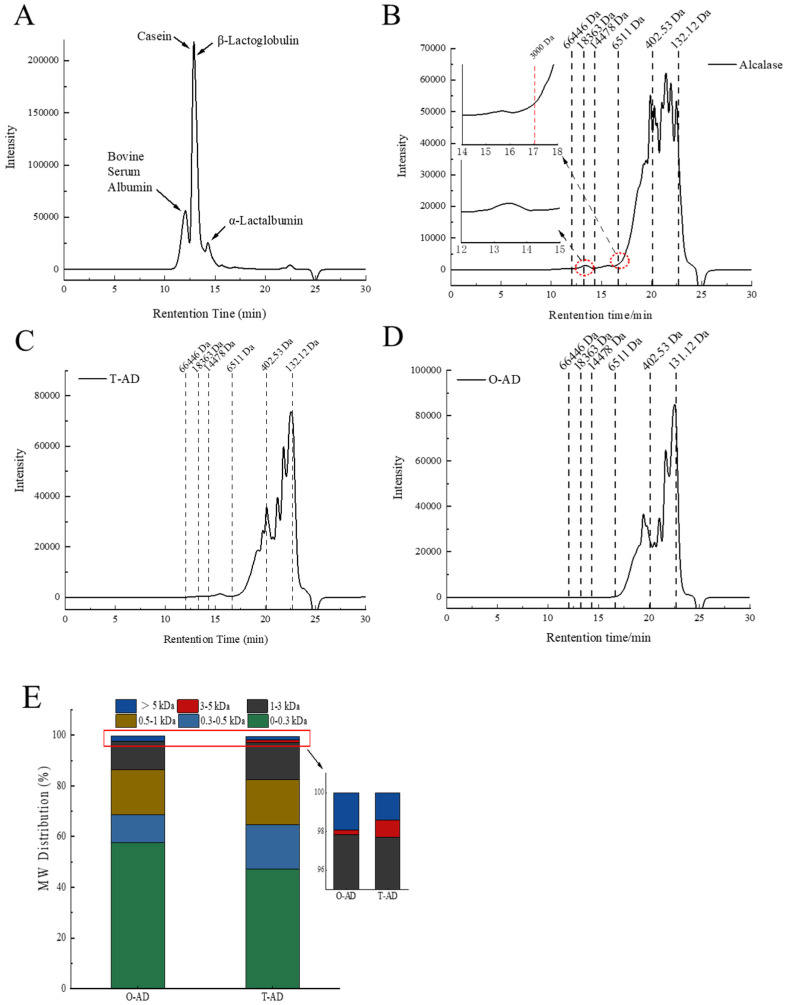
The molecular weight distributions of WPI and WPHs. (**A**) milk protein; (**B**) hydrolysates of alcalase; (**C**) T-AD; (**D**) O-AD; (**E**) distribution of the molecular weight of the O-AD and T-AD. 0–0.3 kDa, 0.3–0.5 kDa, 0.5–1 kDa, 1–3 kDa, 3–5 kDa, >5 kDa.

The distribution of MWs in WPH samples can be accurately determined using volume exclusion chromatography. As shown in Figure 2A, WPI detected multiple peaks in a molecular weight range greater than 6511 Da for BSA, casein, β-Lg, and α-La, which indicated that the untreated WPIs were all large-molecule proteins (mainly whey proteins). The peak time was delayed to 16.8 min after alcalase hydrolysis, and only a few faint peaks were detected in a molecular weight range above 6511 Da, as shown in Figure 2B, indicating that the larger molecular weight peptides were still present after alcalase hydrolysis. In Figure 2C,D, there is a large difference in the chromatographic peaks of the hydrolysate of the same enzyme after different hydrolysis treatments. The two hydrolysates showed different retention times and wider peaks in a molecular weight range below 6511 Da, indicating a complex composition of low molecular weight peptides in the hydrolysates. This also confirmed the large differences in the hydrolysate of different hydrolysis methods.

The MW distributions of the hydrolysates of the WPI are shown in Figure 2E. The hydrolysates of the O-AD (57.71%) contained significantly higher amounts of peptides with MWs < 0.3 kDa than the T-AD (47.22%) (*p* < 0.05). Consistent with the research results of the chromatogram (Figure 2B–D), hydrolysates greater than 5 kDa were determined in all three hydrolysates. The content of hydrolysates with MWs > 5 kDa in alcalase (4.77%) was significantly higher than the O-AD (1.91%) and T-AD (1.41%). This result indicated that there are significant differences in the composition and content of peptides in the hydrolysates of different enzymatic hydrolysis methods. Therefore, the identification of peptide segments in their hydrolysates is of great significance. Generally, peptides containing 2–20 amino acid residues are bioactive [22]. The results of the present study show that the peptide lengths in WPHs were all less than 20 amino acid residues. Therefore, it is necessary to understand the bioactivity and sensitization of hydrolysates under different hydrolysis methods, which can provide new ideas for the application of milk protein in peptide-based foods.

### 3.3. Peptidomics

**Figure 3:**
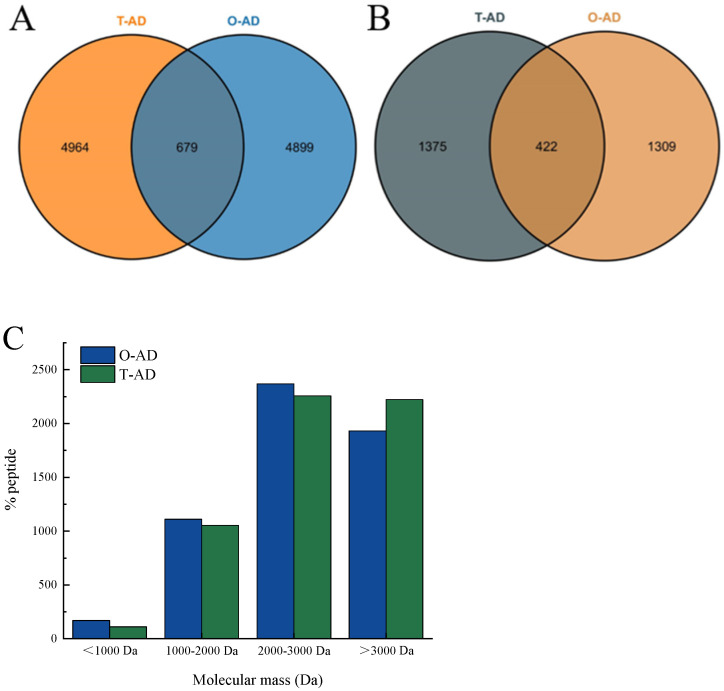
Venn diagram showing the type of peptide composition in WPHs. (**A**) peptides identified in the T-AD and O-AD; (**B**) master access proteins of the T-AD and O-AD; (**C**) molecular mass distribution of peptides identified in the T-AD and O-AD.

The Venn diagram in Figure 3 represents the master proteins of the peptides identified in the WPHs. The results revealed 5643 and 5587 peptides identified in the T-AD and O-AD, respectively, which belonged to 1787 and 1731 master access proteins, respectively. Furthermore, 679 common peptides were detected in all hydrolysates, belonging to 422 master access proteins. Interestingly, 4964 independent peptides were identified in the T-AD, belonging to 1375 master access proteins (Figure 3A). In the O-AD, 4899 independent peptide segments were identified, belonging to 1309 master access proteins. Analysis of the MWs of the identified peptides showed that the proportion of peptides was higher in the O-AD than in the T-AD within an MW range of 2000–3000 Da, while the proportion in the T-AD was significantly higher than that in the O-AD in an MW range of >3000 Da. The difference in peptide content indicated that different hydrolysis methods could affect the degradation process of WPI. The results of the peptide composition and master access protein indicated that different enzyme hydrolysis methods were important factors influencing the composition of peptides. This variation in peptide composition could potentially result in differences in the allergenicity and bioactivity of the hydrolysate residues, among other properties. Therefore, analyzing the composition and differences between milk-derived protein peptides and their respective master access proteins is of great significance. The differences in the number of peptides identified in the hydrolysate can be visualized in the Venn diagram, but it is not possible to directly observe the differences in protein composition and peptide distribution among different WPI hydrolysates [26].

**Figure 4:**
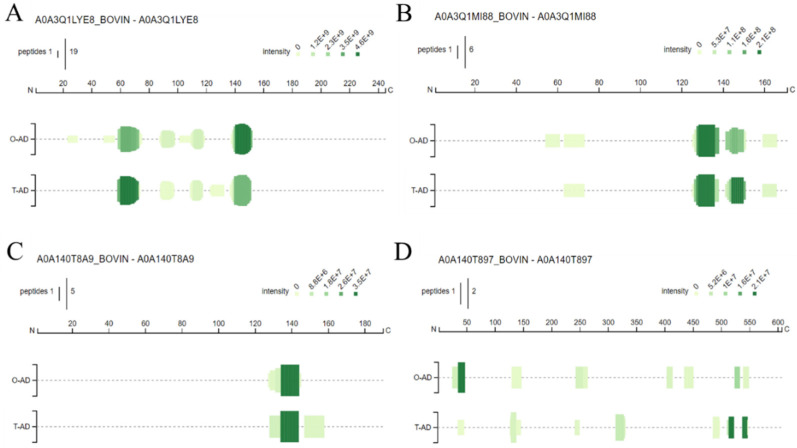
Peptide profiles of the identified peptides of WPHs, including bovine progestagen-associated endometrial protein (A0A3Q1LYE8) (**A**), Shisa family member 6 (A0A3Q1M188) (**B**), bovine κ-casein (A0A140T8A9) (**C**), and albumin (A0A140T879) (**D**). The height and intensity of the green bars correspond to the count of peptides and the sum of the peptide intensities overlapping this position.

Peptide profiles are graphical representations of information on peptides identified via mass spectrometry. They include data on amino acid coverage, the proteins to which they belong, overlap rates, and their relative abundance [27]. The peptide profiles of WPHs are illustrated in Figure 4, mainly including the Progestagen-associated endometrial protein (A0A3Q1LYE8), Shisa family member 6 (A0A3Q1M188), κ-casein (A0A140T8A9), and albumin (A0A140T897). The results of volume exclusion chromatography indicated that the MW distribution of the two hydrolysates was similar, indicating that the hydrolysates contained a significant amount of milk protein fragments. Figure 4A depicts that, among the bovine domain-containing proteins, the O-AD had the most abundant peptide composition compared with the T-AD, such as multiple independent peptide segments at amino acid sequences AA (20–30) and AA (100–110). The amino acid sequence of the T-AD hydrolysis product was detected as a separate peptide near AA (120–140). In α-La (Figure 4B), the O-AD hydrolysate amino acid sequence was detected as a separate peptide near AA (50–60), and the T-AD hydrolysate had the highest relative abundance value, with a response value of 109, indicating that this peptide was the most abundant in the hydrolysis product. In bovine albumin (Figure 4D), both samples were identified with rich peptide information, with lower coverage of the hydrolysis product of the T-AD in the peptide profile compared with the O-AD. This might be due to the differences in peptide composition and abundance values of the hydrolysates of WPI proteins between different hydrolysis methods.

Table 1 presents details on the peptide composition and other data derived from the detection of the WPH. The two hydrolysates identified by LC-MS/MS comprised the main coverage, number of peptide sequences, total number of peptide spectrum matches (PSMs), and the HT score sequence of bovine domain-containing protein, α-La, bovine κ-casein, bovine β-casein, and bovine albumin. Coverage refers to the percentage of sequences covered by identifiers in the included search. The coverage of the O-AD hydrolysates was the highest in the following proteins: Shisa family member 6 (A0A3Q1M188) with 29%, Progestagen-associated endometrial protein (A0A3Q1M701) with 38%, bovine β-casein with 15% (A0A452DHW7), and bovine albumin (A0A140T897) with 18%. Peptides refer to the total number of different peptides identified. Interestingly, the T-AD was detected in a higher number of peptides in the albumin (17) protein than in the O-AD (12). The main reason for this difference is that Protease A 2SD had both endopeptidase and exopeptidase activities and a wide range of cleavage sites. Furthermore, this might result in the recognition of more peptides when hydrolysis occurs at the ends of the peptide. Therefore, a higher content of specific peptide segments was found in its hydrolysate. PSMs refer to the number of peptides matched to the secondary spectrum, which is one of the most important indicators in mass spectrometry analysis. A large number of peptides was identified in both hydrolysates (Table 1). This result also indicated significant differences in the results of protein hydrolysis when different enzymes were employed. However, peptides derived from β-Lg were not detected in the hydrolysates of WPI after hydrolysis in different modes, and a combination of the results of volume exclusion chromatography and SDS-PAGE can be used to infer that β-Lg was completely hydrolyzed to amino acids or low molecular weight peptides.

### 3.4. Identification of the Bioactive and Allergen Peptides

#### 3.4.1. Bioactive Peptides

The identified peptides were searched against and selected from the BIOPEP bioactive peptide database. The matching parameters were set so that the amino acid sequences of the identified peptides overlapped exactly with those in the database. The results are depicted in Table 2. Five bioactive peptides were identified in the BIOPEP database, each with ACE and DPP IV inhibitory activities. Four bioactive peptides were identified for each of the two hydrolysates, and three of these peptides were identified in all samples. The amino acid sequence of the first peptide was VLDTDYK, which belonged to bovine whey protein (AA, 94–100) and had high ACE activity, with an IC_50_ value determined by Pihlanto-LeppÄLÄ et al. [28] to be 964 μM. The second peptide amino acid sequence identified was TPEVDDEALEK, which belonged to β-Lg (AA, 125–135) and exhibited DPP IV inhibitory activity (i.e., an IC_50_ of 319.5 μM) [29]. The third peptide amino acid sequence identified was DKVGINY, which belonged to the α-La amino acid sequence of 97–103, exhibiting ACE-inhibitory activity (i.e., an IC_50_ of 99.9 μM) [30]. As depicted in Table 2, the MWs of the bioactive peptides identified in the hydrolysates ranged from 807.39 to 1323.71 Da. This result confirmed that low-MW peptides exhibited significant bioactivity compared with their high-MW peptides or intact proteins [31].

#### 3.4.2. Allergen Peptides

As depicted in Table 3, 15 peptides with allergenic properties were identified in the stepwise hydrolysates by accessing the BIOPEP database. Furthermore, 12 of these allergenic peptides were derived from β-Lg, 2 from α-La, and 1 from αs2-casein. Moreover, 12 peptides were identified in both hydrolysis products, of which 9 were total allergenic peptides, including 7 peptides of the major allergenic protein β-lactoglobulin and 1 peptide each of α-La and αs2-casein. Three specific sensitizing peptides were identified in the O-AD sample, all of which belonged to β-Lg. Three specific allergenic peptides were identified in the T-AD sample, of which two belonged to β-Lg and one belonged to α-La. Nine allergenic peptides were identified in the two samples: YQEPVLGPVRGPFP (β-casein, AA: 243–256), TPEVDDEALEK (β-casein, AA: 140–151), DIPNPIGSEN (αS1-casein, AA: 196–205), FSDIPNPIGS (αS1-casein, AA: 194–203), and HQPHQPLPPT (β-casein, AA: 195–204). These results were consistent with the findings of Sharma et al. [32] in sensitization research on milk proteins. The results indicated that the minimum MW of immunoglobulin E (IgE) and whey protein binding ranged from 970 to 1400 Da. Van, Meijer, and Schmidt [33] demonstrated that epitopes of β-Lg and α-La were still present in hypoallergenic infant formulations with high DH. The MWs of the allergenic peptides identified in the hydrolysates ranged from 817.41 to 1273.59 Da (Table 3), indicating that the small-molecule hydrolysates also retained some allergenic epitopes. The results of the bioinformatics comparison also indicated the peptide profiles of the hydrolysates from different hydrolysis methods.

### 3.5. Functional Properties of WPH Samples

#### 3.5.1. Antioxidant Activity

**Figure 5:**
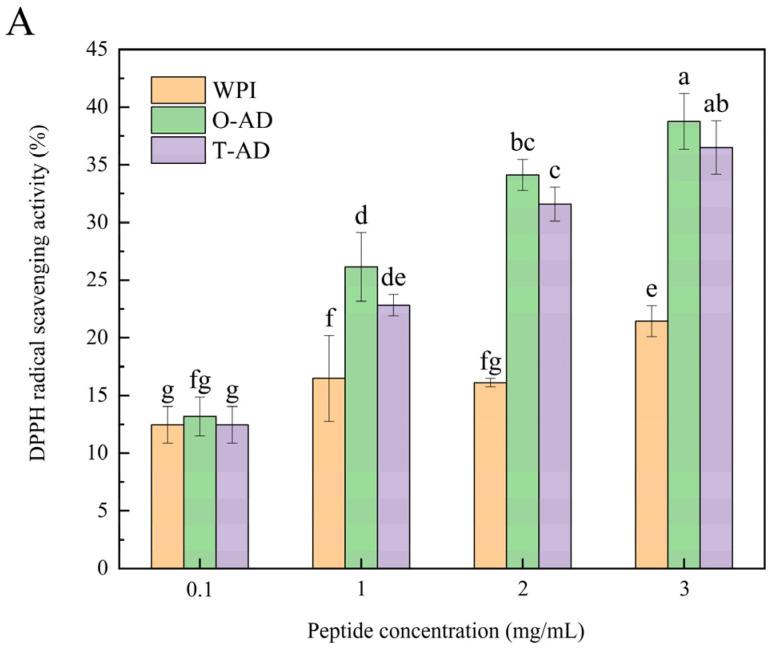

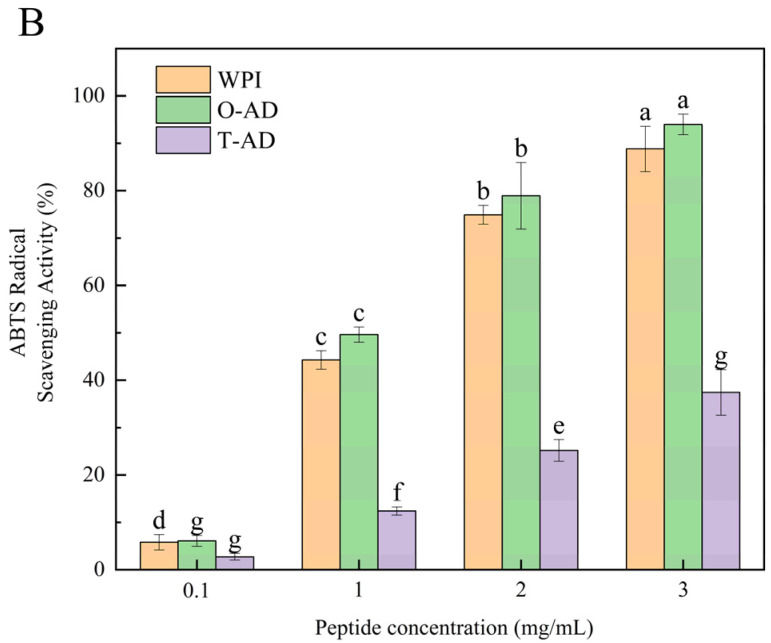
Antioxidant activities of WPI and WPHs. (**A**) DPPH radical scavenging activity; (**B**) ABTS radical scavenging activity. All data are mean ± S.D. values. Different letters indicate significant differences between samples of different concentrations (*p* < 0.05).

The simplest, most effective, and most commonly used method to produce bioactive biological peptides from proteins is enzymatic hydrolysis with protease [34]. Figure 5A shows the scavenging activity of WPI hydrolysis products on DPPH free radicals following different hydrolysis methods. In this study, the combination of protease with exopeptidase and endopeptidase activities could effectively improve the antioxidant capacity of hydrolysates. The results shown in Figure 5A indicated that the DPPH radical scavenging activity of the hydrolysates significantly increased with an increase in the concentration of protein hydrolysates (*p* < 0.05). When the concentration of WPI and two hydrolysates was 3 mg/mL, their DPPH radical scavenging activity peaked at 21.44% (WPI), 38.76% (O-AD), and 36.49% (T-AD). Arise et al. [35], examining peanut protein hydrolysates from Bambara, found that peptides with lower MWs exhibited higher DPPH radical scavenging activity. A significant difference was observed in DPPH radical scavenging activity between the two different hydrolysis methods. A higher DPPH radical scavenging activity was observed using the one-step hydrolysis method. This could be because both enzymes acted simultaneously on the substrate protein during one-step hydrolysis, resulting in more complete degradation of the protein and the production of smaller peptides with higher biological activity. Additionally, small-molecule peptides were identified in the O-AD sample and had a relatively high abundance value, whereas they were not identified in the T-AD sample. These antioxidant peptides might lead to the differences in DPPH radical scavenging activity. The deep hydrolysis products, owing to their strong DPPH scavenging properties, could serve as valuable ingredients for preventing oxidative deterioration.

ABTS radical scavenging activity has been widely used as a determining factor for antioxidant activity [36]. The antioxidant activity of the peptides is influenced by the amino acid sequence and length of the peptides produced by protein hydrolysis. Figure 5B shows the ABTS radical scavenging activity of the hydrolysates achieved using different hydrolysis methods. The results revealed the concentration of WPI and hydrolysates as 3 mg/mL, and the ABTS radical scavenging activity was in the order O-AD (93.99%) > WPI (88.81%) > T-AD (37.41%). The results indicated that hydrolysates might be a useful component in preventing free radical damage in biological systems. Meanwhile, the results indicated that the ABTS radical scavenging activity of the O-AD and T-AD hydrolysates treated using different hydrolysis methods was significantly higher than that of the DPPH radical scavenging activity. This finding is consistent with the results obtained by Liu et al. [36], and this might be due to the loss of low molecular weight peptides and hydrophobic peptides in the hydrolysate after treatments, such as dissolution and centrifugation. Therefore, water-soluble hydrolysates exhibited significant ABTS radical scavenging ability and were less likely to react with lipid-soluble DPPH [37]. Additionally, the O-AD had a high radical scavenging capacity and a high percentage of MWs in a range of 0–500 Da (82.08%). Thus, these small-molecule peptides play a key role in radical scavenging.

#### 3.5.2. Antigenicity

**Figure 6:**
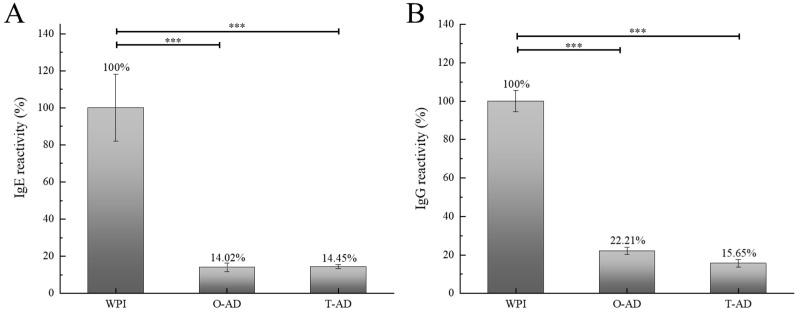
IgE binding (**A**) and IgG binding (**B**) of WPI and WPHs determined by indirect competitive ELISA. ANOVA was used to determine statistical significance, *** *p* < 0.001.

As the degree of degradation of WPI and hydrolysates varies with different hydrolysis methods, so does the variation in the reduction degree of antigenicity. Compared with WPI, the IgE-binding capacity of WPHs was significantly reduced. As illustrated in Figure 6A,B, the binding capacity of specific antibody IgE of the hydrolysate was reduced by 85.98% (O-AD) and 85.55% (T-AD). The IgE-binding capacity of the O-AD sample was the lowest (14.02%). The enzyme-linked immunosorbent assay results indicated a significant reduction in the antigenicity of WPHs (*p* < 0.001). Whey proteins and casein are the major allergens in milk proteins, and β-Lg is the most important allergen in milk proteins of species other than human breast milk [38]. The results of the IgE binding capacity indicated that one-step and two-step hydrolyses of WPI could significantly reduce and eliminate the allergenicity of the obtained peptide. The results of the IgG binding capacity of WPHs are illustrated in Figure 6A,B. The IgG binding capacity decreased by 77.79% (O-AD) and 84.35% (T-AD). Compared with the O-AD sample, the binding capacity of the specific antibody IgG of the T-AD sample was significantly reduced (*p* < 0.001). This might be because the residual linear epitopes of the one-step hydrolysis method might trigger an allergic reaction, in addition to the conformational (nonlinear) epitopes that complicate the IgG binding capacity. Table 3 presents three unique peptides in the O-AD sample (RTPEVDDEALE, TPEGDLEILL, and PTPEGDLEIL) that belonged to β-Lg, with a high abundance value. These unique peptides might lead to differences in allergic reactions. Therefore, the IgG binding capacity of the hydrolysates was different. Huby, Dearman, and Ian [39] reported that cross-linking of FcεRI required the allergen to contain at least two IgE-binding epitopes and that the epitope could theoretically extend to 15 amino acid residues. Therefore, the smaller the molecular weight of the peptides in the milk protein hydrolysate, the lower the allergic reactivity. The results of the IgE and IgG binding capacities revealed that a lower level of antigenicity still remained in the extensive hydrolysate.

## 4. Conclusions

Bioinformatics and peptidomics were used to identify the peptide sequences and potential functional peptides of hydrolysates under different hydrolysis modes. The distribution of the MWs of peptides was found to be similar across different hydrolysis methods. However, peptide profiles and peptide information indicated significant differences. This implies that the hydrolysis methods used had significant effects on the hydrolysis results of the same protein. Furthermore, 5643 and 5587 peptides were identified in the T-AD and O-AD samples, respectively. Measurements of the DPPH and ABTS scavenging activities confirmed that the O-AD had a high free radical scavenging capacity, and hydrolysates might be a useful component in preventing oxidative degradation. The results of the IgE and IgG binding capacities indicated that one- and two-step hydrolyses of WPI can significantly reduce and ideally eliminate the allergenicity of the obtained peptides. This study provides novel ideas for optimizing hydrolysis methods and targeting the application of WPHs in foods.

## Figures and Tables

**Table 1:** Number of peptides and coverage in WPHs of Shisa family member 6, albumin, β-casein κ-casein, and progestagen-associated endometrial protein as identified by LC-MS/MS.

Protein	Protein Groups	O-AD	T-AE
Shisa family member 6 A0A3Q1M188	Coverage [%]	29	27
	# Peptides	16	14
	# PSMs	67	64
	Score Sequest HT	158.93	149.23
Albumin A0A140T897	Coverage [%]	18	15
	# Peptides	12	17
	# PSMs	25	43
	Score Sequest HT	70.25	115.88
κ-casein A0A140T8A9	Coverage [%]	9	14
	# Peptides	5	3
	# PSMs	16	21
	Score Sequest HT	37.79	53.33
β-casein A0A452DHW7	Coverage [%]	15	5
	# Peptides	4	1
	# PSMs	6	1
	Score Sequest HT	15.36	2.2
Progestagen-associated endometrial protein A0A3Q1M701	Coverage [%]	38	32
	# Peptides	59	48
	# PSMs	506	378
	Score Sequest HT	1508.64	1086.96

**Table 2:** Identification of the major peptides in WPHs.

Amino Acid Sequence	O-AD	T-AE	Mass (Da)	Reported Bioactivity	Start–End Position	Protein Accession
VLDTDYK	√	√	852.92	ACE inhibitor	[94–100]	P02666
TPEVDDEALEK	√	√	1244.56	DPP IV inhibitor	[125–135)	A0A3Q1LYE8
DKVGINY	√	√	807.39	ACE inhibitor	[97–103]	A0A3Q1MI88
YPFPGPIPN	√		1000.48	ACE inhibitor	[75–83]	P02666
LKPTPEGDLEIL		√	1323.71	DPP IV inhibitor	[62–73]	A0A3Q1M701

The peptide with reported bioactivity in BIOPEP (http://www.uwm.edu.pl/biochemia/index.php/pl/biopep, accessed on 24 June 2021, 4321 entries).

**Table 3:** Identification of the major allergen peptides in WPHs.

Amino Acid Sequence	O-AD	T-AE	Allergen	Protein	A *	Start–End Position	Mass (Da)
TKLTEEEKNR	√	√	Bos d 8 alpha s2	αS2-casein	0.8468	[166–175]	1247.65
TPEVDDEALEK	√	√	Bos d 5 beta-lg	β-lactoglobulin	1.7022	[141–151]	1245.58
EVDDEALEKF	√	√	Bos d 5 beta-lg	β-lactoglobulin	1.7022	[143–152]	1194.55
LDDDLTDDIM	√	√	Bos d 4	α-lactalbumin	0.4648	[129–138]	1165.49
RTPEVDDEAL	√	√	Bos d 5 beta-lg	β-lactoglobulin	1.7022	[140–149]	1144.54
TPEVDDEALE	√	√	Bos d 5 beta-lg	β-lactoglobulin	1.7022	[141–150]	1117.48
AEKTKIPAVF	√	√	Bos d 5 beta-lg	β-lactoglobulin	1.7022	[89–98]	1103.64
VEELKPTPEG	√	√	Bos d 5 beta-lg	β-lactoglobulin	1.7022	[59–68]	1098.56
TDYKKY	√	√	Bos d 5 beta-lg	β-lactoglobulin	1.7022	[113–118]	817.41
RTPEVDDEALE	√		Bos d 5 beta-lg	β-lactoglobulin	1.7022	[140–150]	1273.59
TPEGDLEILL	√		Bos d 5 beta-lg	β-lactoglobulin	1.7022	[65–74]	1099.58
PTPEGDLEIL	√		Bos d 5 beta-lg	β-lactoglobulin	1.7022	[64–73]	1083.55
ELKPTPEGDL		√	Bos d 5 beta-lg	β-lactoglobulin	1.7022	[61–70]	1098.56
ENSAEPEQSL		√	Bos d 5 beta-lg	β-lactoglobulin	1.7022	[124–133]	1103.48
KFLDDDLTDD		√	Bos d 4	α-lactalbumin	0.4648	[127–136]	1196.53

* Frequency of the occurrence of all epitopes in a protein “A”. The peptide with reported bioactivity in BIOPEP (http://www.uwm.edu.pl/biochemia/index.php/pl/biopep, accessed on 24 June 2021, 4321 entries).

## Data Availability

The original contributions presented in the study are included in the article and Supplementary Material, further inquiries can be directed to the corresponding author.

## Supplementary Materials

The following supporting information can be downloaded at: https://www.mdpi.com/article/10.3390/foods13182978/s1, Figure S1. Method flowchart; Table S1. Information on milk protein allergy patients.
