# Supplementary material for: Characterization of Peptide Profiles and the Hypoallergenic and High Antioxidant Activity of Whey Protein Hydrolysate Prepared Using Different Hydrolysis Modes
Source: Foods. 2024 Sep 20;13(18):2978. doi: 10.3390/foods13182978 (PMC11431592; doi:10.3390/foods13182978)
Supplement: Supplementary file 1 [file foods-13-02978-s001.zip › foods-3183200-supplementary.pdf]

Table Captions

Table S1. Information of milk protein allergy patients.

Table S1. Information of milk protein allergy patients.

Number	Gender	Age	Diagnosis	Total IgE level（IU/mL）	Specific IgE level（IU/mL）
1	female	67y	Allergic rhinitis, Multi-allergic	1220	＞＝100
2	female	67y	Allergic rhinitis, Multi-allergic	ND[*]	73.9
3	male	32y	Allergic asthma	379	23.8
4	male	28y	Allergic rhinitis	449	87.2
5	male	20y	Allergic rhinitis, Multi-allergic	1736	＞＝100

ND[*]: Not detected

Figure Captions

Figure S1. Method flowchart.

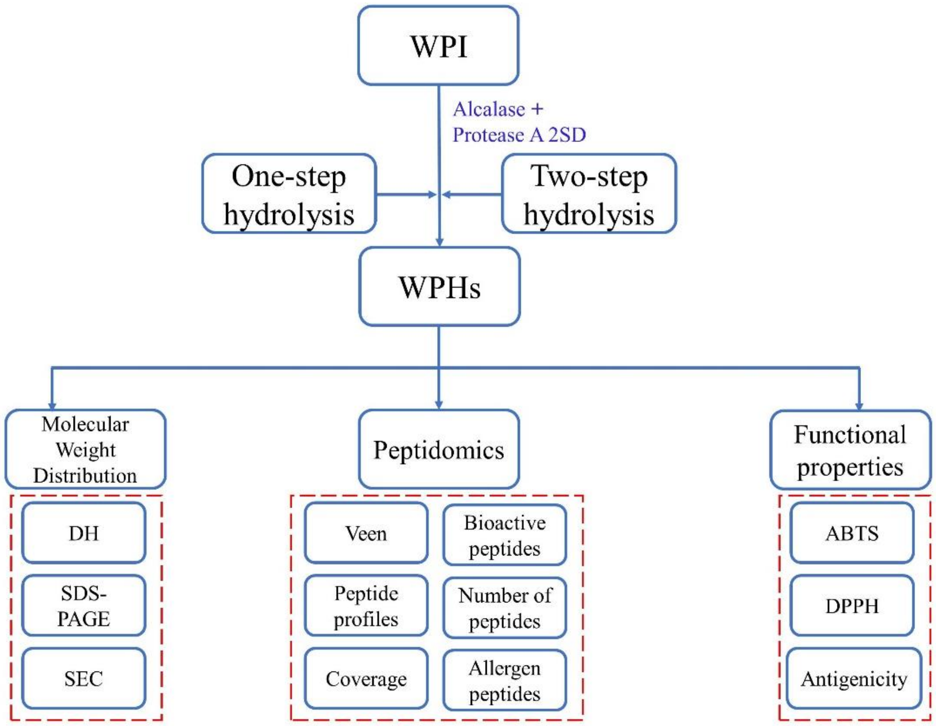

Figure S1. Method flowchart.
